# Implementation, Validation and Clinical Testing of Oximetry Device for Microcirculation Assessment in Oral Tissue

**DOI:** 10.3390/s25216604

**Published:** 2025-10-27

**Authors:** Hojat Lotfi, Bibiana Falcão, Valentina Vassilenko

**Affiliations:** 1 Laboratory for Instrumentation, Biomedical Engineering and Radiation Physics (LibPhys-UNL), NOVA School of Science and Technology, NOVA University of Lisbon, Campus FCT-UNL, 2896-516 Caparica, Portugal; h.lotfi@campus.fct.unl.pt (H.L.); bibianafalcao@gmail.com (B.F.); 2 Clínica Dr. Alexis Mendonça, Rua Visconde da Luz, 12 1º, 2750-414 Cascais, Portugal; 3 NMT, S.A., Edifício Madan Parque, Rua dos Inventores, 2825-182 Caparica, Portugal

**Keywords:** oximetry, photoplethysmography, device development, gingiva perfusion, microcirculation, dental implant

## Abstract

The recent rise in living standards has been accompanied by increased awareness and emphasis on oral health. Non-invasive assessment of gingival microcirculation and accurate evaluation of oxygen supply to oral tissues are critical for the early diagnosis of oral diseases. These factors also play a pivotal role in optimizing treatment planning and improving outcomes in dental implantology. In this study, we report the development and implementation of a novel pulse oximetry device based on reflective photoplethysmography technology, designed for non-invasive, real-time monitoring of gingival health through the measurement of oxygen saturation levels. A detailed description of the technology, including key aspects of sensor probe design, is provided, with particular emphasis on the calibration process and performance evaluation of the prototype. Furthermore, we present and discuss the first proof-of-concept gingival oxygen saturation measurements obtained in a clinical setting during oral rehabilitation consultations.

## 1. Introduction

The high global prevalence of oral and dental diseases, combined with limited access to affordable and appropriate healthcare services, underscores the need for innovative methods and tools in dentistry for both prevention and treatment. Periodontal disease represents the second most common dental pathology worldwide and exhibits high prevalence across all age groups, including children and adolescents [1]. This chronic infection, caused by Gram-negative bacteria such as Porphyromonas gingivalis and *Treponema denticola*, impairs gingival perfusion by promoting local inflammation, endothelial damage, and tissue destruction [2,3,4].

Since the 1980s, the widespread adoption of dental implants and advancements in implant technology have significantly improved public health by restoring oral function and aesthetics [5,6]. However, proper maintenance and post-operative care have become essential for ensuring the longevity of dental implants.

The oral tissues contain an extensive vascular network, which plays a critical role in healing processes, including recovery of inflammation, periodontal disease management, implant placement, and osseointegration [7,8]. The high vascularity of oral tissues explains the profuse bleeding associated with oral wounds or trauma, but it also contributes to their remarkable healing potential [8]. Oxygen transport through the circulatory system is fundamental for cellular energy production, as hemoglobin delivers oxygen required for glucose metabolism. Without an adequate oxygen supply, tissues cannot sustain normal metabolic functions, leading to cell death and impaired healing [7,9].

In implantology, successful osseointegration depends on several factors, including material biocompatibility, precision of implant site preparation, extent of surgical trauma, and type of loading protocol. However, in the early stage of osseointegration, bone vascularity and the formation of new blood vessels play a crucial role in establishing a strong bone-implant connection [10,11]. Research has shown a proportional relationship between the rate of bone remodeling and vascularization levels. After implant placement, proper tissue repair requires the development of a functional vascular system to deliver oxygen and nutrients while also removing cellular debris for complete healing. Bone remodeling depends on neo-angiogenesis, the process of forming new blood vessels [12]. A reduced blood supply to the bone and soft tissue can compromise bone growth and increase the risk of implant failure [13].

Several factors influence alveolar bone blood flow, including presence of teeth, patient’s age, degree of bone resorption and systemic conditions (e.g., diabetes, cardiovascular diseases) [14,15]. The formation and long-term stability of dental implants depend on a well-developed peri-implant bone vascular network [12,16].

At present, the condition of dental implants is primarily assessed through direct observation and clinical examination of the jawbone. However, the availability of a reliable, non-invasive method to evaluate gingival perfusion during osseointegration could substantially reduce the risk of implant failure, extend the functional lifespan of implants, and minimize the costs, pain, and discomfort associated with re-implantation [17]. A device capable of continuously and non-invasively monitoring gingival blood flow and oxygen saturation would provide valuable insights into peri-implant vascularization, enabling clinicians to optimize treatment strategies and ultimately improve patient outcomes.

In this study, we present the development of a device capable of non-invasively monitoring gingival oxygen saturation, which may provide valuable insights into peri-implant vascularization. While blood flow (perfusion) is also a critical determinant of tissue health, the present work focuses specifically on oxygen saturation as the primary measurable parameter.

## 2. Technology Overview

In recent decades, optically based technologies such as Laser Doppler Flowmetry (LDF) and Photoplethysmography (PPG) with its most widely used variant, pulse oximetry (PO), have been employed for assessing microcirculation in oral tissues. Detailed descriptions of the physical principles underlying these technologies, along with their comparative analyses, have been extensively documented in several studies [18,19,20], and will not be reiterated here.

However, it is important to note that optoelectronic devices and sensors based on these technologies have been predominantly used for tooth pulp vitality testing [21,22,23,24]. In some pilot studies for the gingiva microcirculation assessment and periodontal evaluation, LDF sensor was used as a complementary method of monitoring periodontal inflammation [25]. Despite its potential applications, LDF technology presents several limitations in evaluating gingival microvascular health, including probe size constraints; variability in laser types, calibration constants, and measurement units; limited penetration depth, as LDF primarily assesses superficial blood flow, making it less effective for evaluating circulation within periodontal pockets [17,26]. Given these challenges, our research focused on developing a sensor probe based on pulse oximetry technology, which offers a more practical and effective solution for assessing gingival microcirculation.

Pulse oximetry is a valuable tool used for many decades in medicine for the determination of blood oxygen saturation (*SpO*_2_), which has vital importance for living cells and tissues and is one of the key health indicators. Oxygen supply is related to the two main forms of the oxygen-transport protein in red blood cells: oxygenated hemoglobin (oxy-hemoglobin, *HbO*_2_) and deoxygenated hemoglobin (deoxyhemoglobin, *Hb*). An oxygen saturation level of a patient’s blood, *SpO*_2_, is a measure of peripheral capillary oxygen saturation, represented as a percentage of the amount of oxyhemoglobin to total hemoglobin:

(1) $$SpO_{2}=100\times \frac{{cHbO}_{2}}{\left({cHbO}_{2}\;+\;cHb\right)}$$

where *cHbO*_2_, and *cHb* are the concentrations of oxyhemoglobin and deoxyhemoglobin, respectively [27].

**Figure 1:**
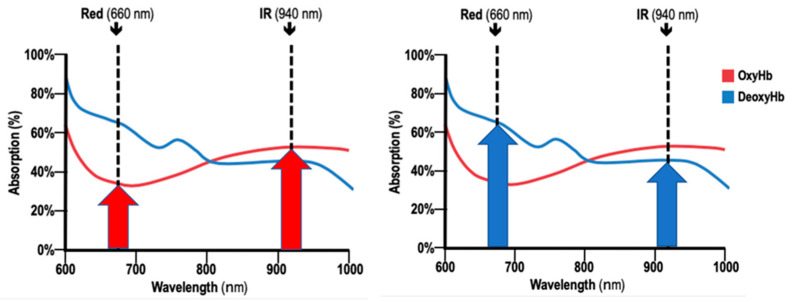
Oxyhemoglobin and deoxyhemoglobin absorption in different wavelengths (adapted from [33]).

A detailed theory of pulse oximetry and noninvasive *SpO*_2_ measurement, both from a theoretical point of view and in terms of technological findings, as well as its application in medicine, can be found elsewhere [28,29,30,31,32]. In general, pulse oximetry is based on two physical principles:

1. Modulation of the incident light passing through the tissue by absorption of pulsatile arterial blood forming the photoplethysmography (PPG) signal. The PPG signals consist of an AC component representing the light absorbed by the pulsatile arterial blood, and DC component that captures the effects of light absorbed by venous and other non-pulsatile blood and tissue components, such as bone, water, etc. [28].
2. Different absorption characteristics of oxyhemoglobin and deoxyhemoglobin for different wavelengths of incident light. The Beer Lambert’s law, in which the absorption depends on the concentration and optical properties of the molecule compound of tissue through which the light at a certain wavelength is traveling, allows us to measure *SpO*_2_ by using the molar extinction coefficients of *HbO*_2_ and *Hb* [29]. Oxygenated hemoglobin or oxyhemoglobin, *HbO*_2_, absorbs more infrared light and less red light than deoxygenated hemoglobin, *Hb*. Figure 1 shows the absorption of *HbO*_2_ and *Hb* at a red light (660 nm wavelength) and infrared light (940 nm wavelength) of commonly used LEDs in pulse oximetry [30].

Since the pulsatile, AC, and non-pulsatile, DC, components of the received PPG signals are different for different LED wavelengths, the modulation ratio *R* of light absorbance at the two wavelengths *λ*_1_ and *λ*_2_, can be calculated by the following equation:

(2) $$R=\frac{AC_{\lambda_{1}}/DC_{\lambda_{1}}}{AC_{\lambda_{2}}/DC_{\lambda_{2}}}$$

where *AC_λ_*_1_ and *AC_λ_*_2_ are the *AC* amplitudes of the red and infrared signals, respectively, and the *DC_λ_*_1_ and *DC_λ_*_2_ are the *DC* offsets of the red and infrared signals, respectively [28,33]. Finally, the *SpO*_2_ values are calculated through the utilization of empirically derived calibration curves based on the modulation ratio *R* [34] or via a lookup table with empirical values based on healthy patients’ measurements [35].

Pulse oximeter sensors generally have a combination of at least two or more emitting diodes (LEDs) at different wavelengths and one photodiode that receives the modulated PPG signal and transmits the received information to the microprocessor [36]. Low-cost oximeters are usually enough for only a couple of wavelengths, which typically are 660 nm (red) and 940 nm (infrared).

**Figure 2:**
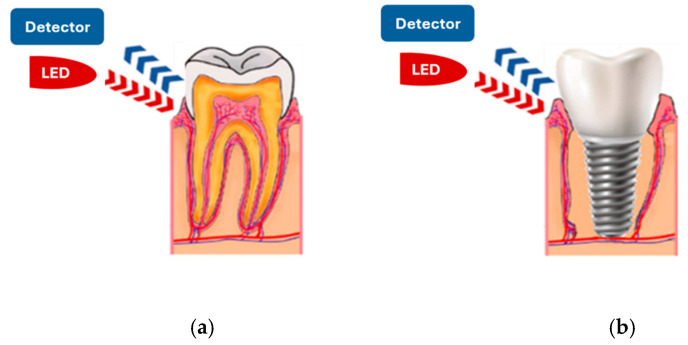
Reflective pulse oximetry sensor applied to gum tissue microcirculation assessment for (**a**) teeth and (**b**) implants.

Depending on the application, the pulse oximetry sensor probe can be designed in a transmissive and reflective working mode. In transmissive pulse oximetry, the photodiode and the LED are placed on opposite sides, so the photodiode collects the residual light that passes through the human tissue [28,37]. In reflective pulse oximetry, the photodiode and the LED are on the same side. The photodiode collects the light reflected from various depths underneath the surface of the skin or other tissue [34,38]. It is important to note that reflective mode pulse oximetry can be applied to various measurement sites, leading to a continuous increase in its demand. To achieve our goal of measuring oxygen saturation in the gums of living teeth or implants (Figure 2), we selected pulse oximetry in reflective mode to develop the sensor probe.

The use of reflective pulse oximetry in the oral cavity offers several advantages but also presents some challenges. One of its key benefits is the ability to position both LEDs and the photodiode in close proximity, allowing for a compact and user-friendly design. This makes the sensor probe more practical for use in a dental environment. Additionally, a major source of interference in PPG signals—ambient light—is significantly reduced in the oral cavity. This is because the soft tissues of the mouth naturally attenuate certain wavelengths of ambient light, minimizing external signal disruptions and improving measurement accuracy.

## 3. Device Development

The implementation of a pulse oximeter requires the development of multiple interconnected components. In this multi-component system, information flows sequentially from one section to the next, with the output of each component serving as the input for the next stage. This structured process ensures the creation of a fully functional device. The optimal performance of the final instrument depends on the proper functioning of each individual component. Any malfunction at one stage can compromise the accuracy and reliability of the entire system.

**Figure 3:**
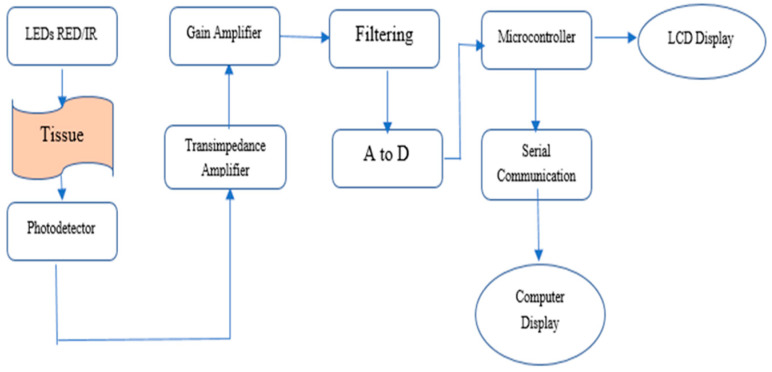
Pulse oximeter device block diagram.

The block diagram illustrating the architecture of the pulse oximeter developed in this study is presented in Figure 3.

Technology and sensor design integrate electronic, software, and mechanical components, each contributing specific functionalities to ensure optimal performance [34,38,39,40,41].

### 3.1. Hardware Development and Probe Design

The hardware design of the pulse oximeter is structured into five main components:

- Sensor Probe: Consists of two dual light-emitting diodes (LEDs) and a photodiode as a light detector.
- Signal Amplifier and Noise Filter: Enhances signal clarity by reducing interference.
- Microcontroller: Processes the data and controls the device’s operation.
- Power Supply: Ensures stable energy delivery to all components.
- Display Unit: Provides real-time visualization of the measurement results.

Advancements in pulse oximetry technology and component design have significantly enhanced the performance quality of new-generation pulse oximeters. The main improvements include: (i) Increased photocurrent, leading to better sensor responsiveness; (ii) Enhanced contrast, improving signal clarity; (iii) Higher signal-to-noise ratio, reducing interference and improving measurement accuracy.

The design of the prototype probe required the careful selection of high-sensitivity optoelectronic components to ensure accurate *SpO*_2_ measurement. Because pulse oximetry relies on the differential absorption spectra of hemoglobin, two wavelengths were employed: 660 nm (red light), primarily absorbed by deoxygenated hemoglobin (*Hb*), and 940 nm (infrared light), preferentially absorbed by oxygenated hemoglobin (*HbO*_2_). To achieve reliable performance in reflective mode, ultra-bright LEDs capable of delivering stable high-intensity output were paired with a photodiode optimized for near-infrared detection. This configuration was chosen to maximize the detection of small pulsatile changes in gingival microcirculation and improve overall measurement robustness.

The optical sensor unit incorporated one red LED (OSRAM SFH 2701, λ = 660 nm, forward voltage ~2.0 V, typical operating current 20 mA, radiant intensity ~1.6 mW/sr at 20 mA) and one infrared photodiode (OSRAM SFH 2703, λ = 940 nm, active area 7 mm^2^, dark current ≤2 nA, responsivity ~0.6 A/W at 940 nm). These components were selected for their stable optical characteristics, low power consumption, and suitability for portable pulse oximetry applications. The average current consumption of LED during operation was approximately 20 mA.

The distance between the LED and photodiode was fixed at 5 mm, representing a balance between penetration depth into gingival tissue and signal-to-noise ratio in reflective-mode pulse oximetry. This optical geometry was determined to be optimal for capturing gingival oxygenation signals while maintaining compact probe design and minimizing measurement noise.

The amplifier and filter system play a crucial role in signal processing due to the low amplitude of signals and the high susceptibility to noise and interference.

The analog-to-digital (A/D) converter used in the system features a high sampling rate and is capable of detecting signals in the millivolt range. However, any superimposed noise can significantly disrupt the accuracy of the readings. To ensure reliable measurements, the desired signals falling within the 0.1–3 Hz range are isolated using two passband filters that effectively remove all frequencies outside the 0.7–3 Hz band. This filtering process minimizes external noise and enhances signal clarity.

The amplifier gain configuration is designed to boost weak signals while maintaining signal integrity. The first amplifier stage provides a gain of approximately 150 and the second amplifier stage further amplifies the signal by a factor of 10. The total system gain is approximately 1500, ensuring sufficient signal strength for accurate processing and analysis.

**Figure 4:**
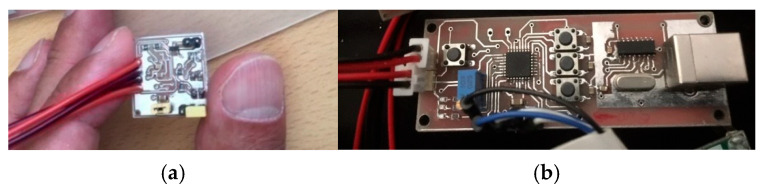
Initial big-size head probe (**a**) and main hardware (**b**) design of reflective pulse oximetry sensor.

The initial prototype of the probe was developed on a 3 × 3 cm square board to evaluate its performance accuracy before proceeding with miniaturization (Figure 4a). To enhance ergonomics and ensure suitability for use in the oral cavity, the final design was optimized to feature dimensions, shape, and mechanical properties similar to those of an electric toothbrush [42].

Due to the limited space on the PCB and the requirement for compact components, a precise and carefully engineered design was necessary. A complete circuit schematic was developed for PCB layout and Gerber file generation. To achieve the smallest possible dimensions, the routing and component placement were meticulously optimized, minimizing trace widths and electrical distances between components and signal paths. As a result, the final PCB measured 10 × 70 mm and featured a double-sided copper coating (Figure 4b).

Regarding the microcontroller selection, it shall be noted that several microcontrollers can be used to implement this probe, provided they meet the required specifications while considering space limitations. One of the most cost-effective options available is the ATmega328, or a similar model equipped with built-in analog-to-digital (A/D) converters, which are essential for sampling and digitizing input analog signals. Among these, the ATmega328P, an AVR microcontroller, is a preferred choice due to its high performance, low power consumption, and suitability for compact design.

The probe was designed to be compact and ergonomic, ensuring easy and safe use in the oral cavity. Special attention was given to material selection, ensuring that all components are non-toxic, non-harmful, and non-allergenic, making them safe for use in direct contact with gums and teeth.

**Figure 5:**
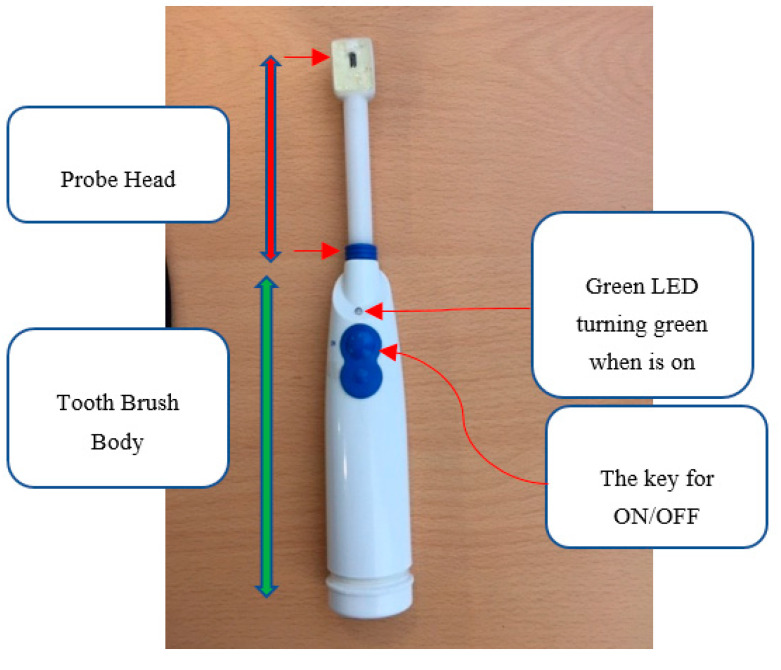
Assembled prototype showing different parts.

The probe head measures 9 × 13 mm, making it well-suited for precise placement in the mouth while maintaining secure contact with the oral tissues. Mechanically, the probe head is firmly connected to the main body, providing high pressure resistance to withstand clinical use. Figure 5 presents the assembled prototype, showcasing its various components. In addition to the ON/OFF control via the user interface, the device includes a built-in hardware switch, enhancing ease of use and allowing dentists to operate it more conveniently during procedures.

To meet the voltage requirements of 5 V and 3.3 V for microcontrollers while ensuring minimal power consumption for safe use in the patient’s mouth, a USB port from PC was chosen as the optimal power source. It is important to mention that powering the device directly from a USB port does not fulfil the IEC 60601-1 safety requirements for medical electrical equipment [43] and, therefore, cannot be considered a final design specification. In this preliminary prototype, USB power was employed only to simplify the experimental setup during feasibility testing, primarily with a battery-operated laptop. This solution also allowed us to maintain a compact probe design while controlling manufacturing costs, making the prototype both practical and cost-effective for early testing. Future iterations of the device will comply with IEC 60601-1 standards through the integration of medical-grade power supplies and isolation measures to ensure safe clinical deployment.

### 3.2. Software Development

Since a single photodiode is used for two LEDs operating at wavelengths of 660 nm and 940 nm, the software must effectively manage the photodiode to handle both light sources. One of the best solutions is to sequentially switch the LEDs ON and OFF, ensuring that only one LED is active at a time, so that the corresponding data can be accurately captured and processed. The steps order for turning ON/OFF the LEDs are as follows:

1. TURN ON the Red LED.
2. WAIT Signal stabilized.
3. READ Red signal.
4. TURN OFF the Red LED.
5. TURN ON IR LED.
6. WAIT Signal stabilized.
7. READ IR signal.
8. TURN OFF IR LED.
9. WAIT Signal stabilized.

The PPG signal represents the light absorbed by the target tissue and consists of two main components: the AC and DC components, as described above. For *SpO*_2_ calculation, the software analyzes the AC and DC components of the PPG signals and determines the modulation ratio, R, of light absorbance, using Equation (2) with wavelengths *λ*_1_ = 660 nm and *λ*_2_ = 940 nm. The oxygen saturation values are then obtained from an empirical table stored in memory, where an R of 3.4 corresponds to 0% *SpO*_2_, and a value of 0.4 represents 100% *SpO*_2_ [35]. Alternatively, the modulation ratio R can be calculated using a well-known method that considers only the intensity of the AC component at two wavelengths [28].

### 3.3. Interface and Display

To enable real-time viewing of oxygen saturation values and data recording, a simple interface was designed to connect the device to a computer via a USB port. To start a measurement, simply click the OPEN key, and to stop it, click the CLOSE key.

**Figure 6:**
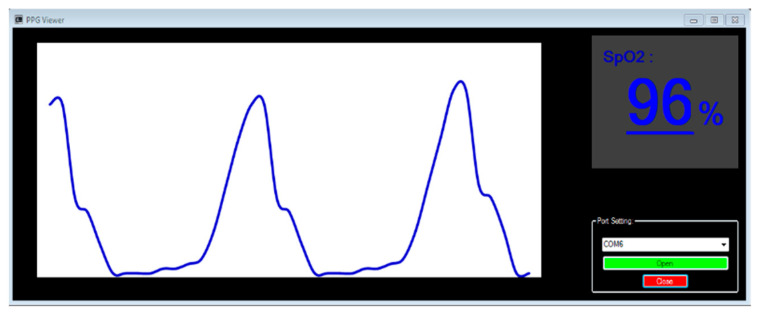
The display interface in a computer screen.

Figure 6 illustrates the interface display, which consists of three main sections:

1. PPG Signal Display Section—Located on the left side of the interface within the white box, this section visualizes the PPG signal in real-time.
2. *SpO*_2_ Value Display Section—Positioned on the right side of the interface, this section displays the oxygen saturation percentage for each measurement.
3. Port Setting Section—Found at the bottom right of the interface, this section allows users to configure the port connection between the device and a computer or laptop to enable data visualization.

The interface was further enhanced with a fourth component—the Audio Signal. This sound, synchronized with the peak of each signal, plays a crucial role for both the user and the patient. It informs the user about the proper operation of the device, reducing the need for continuous visual monitoring. Additionally, it alerts the patient to the device’s activation, potentially improving their cooperation during the measurement process.

## 4. Performance and Calibration Tests

Prototype testing and quality control were conducted throughout all stages of the design process to ensure the accuracy and reliability of the desired performance. Each component of the prototype was individually tested in a laboratory to identify potential issues and resolve any obstacles. Once the individual components were validated, they were assembled into the prototype to create a final product that met the initial design requirements. Following this stage, the prototype underwent further testing to verify its overall performance and ensure compliance with the International Standard requirements of ISO 80601-2-61:2017—Medical electrical equipment, Part 2-61: Particular requirements for basic safety and essential performance of pulse oximeter equipment [41].

To ensure both accuracy and reliability, the performance and calibration of the developed device were verified using two complementary approaches. The first involved testing with a physiological simulator under controlled conditions, which allowed safe and reproducible evaluation of device responsiveness without involving human subjects. The second consisted of in vivo parallel measurements, in which results obtained from the prototype were directly compared with those from a standard, calibrated pulse oximeter. This two-step strategy provided a balanced assessment of the prototype, combining the reproducibility of bench testing with the realism of clinical validation.

The use of a physiological simulator at this stage was chosen to minimize potential risks to human subjects, such as exposure to excessive optical intensity, inadvertent electrical leakage from a non-certified prototype, or local irritation from unvalidated probe materials. By relying on a simulator, preliminary testing of device responsiveness and calibration could be performed safely before proceeding to in-vivo validation.

**Figure 7:**
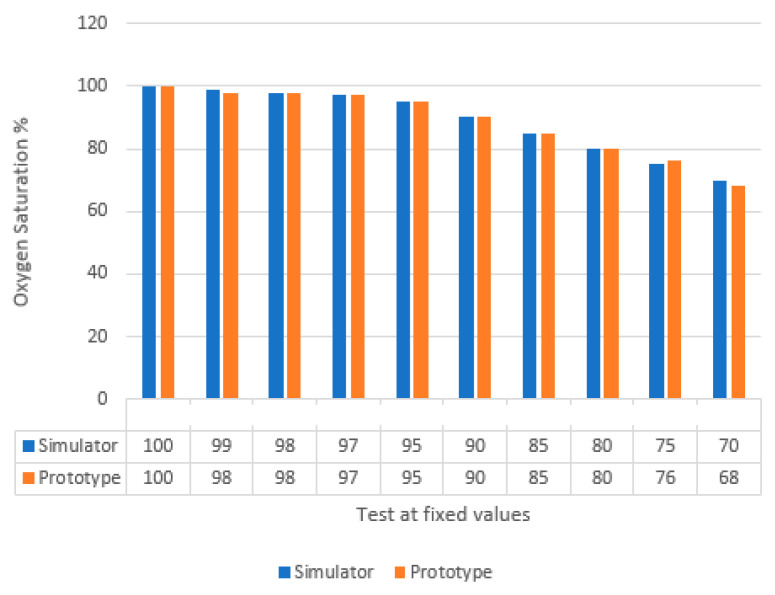
Comparison table between oxygen saturation obtained from prototype against a set value of SPOT LIGHT simulator by Fluke Biomedical Inc.

In pulse oximetry testing, certain simulators can mimic the optical properties of a human finger and its pulsating blood flow. One of the latest simulators can compare up to eight custom R-curves from different manufacturers using a single pulse oximeter [44]. In our study, the SPOT Light simulator by Fluke Biomedical [45] was used exclusively to confirm prototype responsiveness under controlled conditions. In accordance with ISO 80601-2-61:2017 requirements, the test was performed by measuring oxygen saturation levels between 100% and 70% under standardized conditions of 80 BPM, with a maximum allowable standard deviation of ±4.00%. The results, obtained from 10 test points within this range and shown in Figure 7, confirm that all measurements fall within the acceptable limits.

The limitations of simulators must be acknowledged, since they are unable to fully replicate the complex optical properties of gingival tissue, the variability of microcirculation, or systemic factors present in vivo. For these reasons, simulator-based results should be regarded only as a preliminary functional check, and in vivo validation against gold-standard oximetry devices remains necessary to confirm measurement accuracy.

To evaluate the performance of the developed prototypes, we employed a one-to-one comparison method, which is a standard approach widely used in clinical settings for testing medical devices. In this method, the output of the prototype is directly compared with that of a performance-validated reference device. Accordingly, we conducted extensive parallel *SpO*_2_ measurements using finger-based recordings to validate the calibration accuracy and overall performance of our prototype. These readings were compared with those obtained from a clinically validated pulse oximeter OXYM9000 (QUIRUMED), which has a 0–100% measurement range and provides ±2% accuracy for oxygen saturation levels between 70% and 100% [46]. Since the tested prototype device was developed for use in oral tissue, the most critical range for accuracy validation is the 90–100% *SpO*_2_ interval.

The OXYM9000 was selected as the gold-standard comparator in this study because, unlike most commercially available oximeters that operate in transmittance mode, it also functions in reflectance mode. This ensured methodological consistency with our prototype and provided a more appropriate benchmark for evaluation.

All oxygen saturation measurements were performed on arterial blood from the fingers of adult volunteers under controlled experimental conditions of light, temperature, and humidity in the laboratory of NMT, S.A., Portugal. A special protocol was implemented to ensure the accuracy of measurements. According to this protocol:

- Before taking measurements, participants sat in a comfortable position for at least five minutes to achieve a stable heart rate.
- During this period, relevant participant data were collected, including age, sex, skin color, and lifestyle factors such as smoking habits or medical conditions affecting blood circulation (e.g., blood pressure, vasoconstriction, or diabetes).
- All participants provided informed consent before taking part in the study.

This protocol ensured standardized conditions for data collection, enhancing the reliability of the calibration and performance validation process.

According to the protocol, measurements were conducted under stable environmental conditions: the temperature ranged from 22 to 23 °C, humidity from 66% to 72%, and environmental luminosity from 523 to 600 lumens. The time required for oxygen saturation values to appear on commercially calibrated oximetry devices typically varies between individuals, with an average onset of 8 to 10 s after the device is connected. Based on this, the first measurement was taken after 15 s, followed by subsequent measurements at 30 s and 60 s intervals. The process began with the calibrated device, recording heart rate and *SpO*_2_ values at 15, 30, and 60 s after attaching the device to the volunteer’s finger. Immediately afterward, the same procedure was repeated using the prototype device, with *SpO*_2_ values recorded at the same intervals.

**Figure 8:**
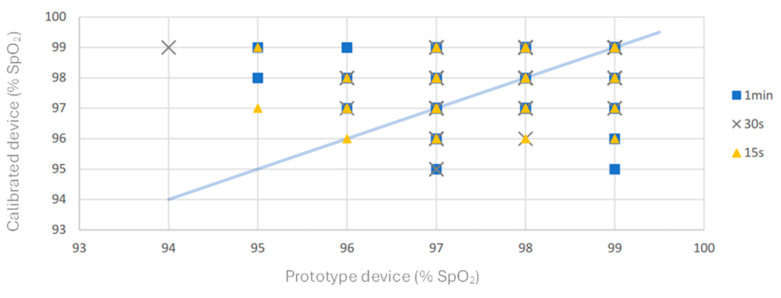
*SpO*_2_ results obtained by the calibrated reference device and prototype for the three-time intervals (15, 30, and 60 s).

A total of 300 *SpO*_2_ measurements within the specified range were obtained from 150 parallel cases, involving male (60%) and female (40%) participants aged 29 to 44 years. Figure 8 illustrates the relationship between the results obtained from our prototype and those from the calibrated reference device across three-time intervals (15, 30, and 60 s).

**Figure 9:**
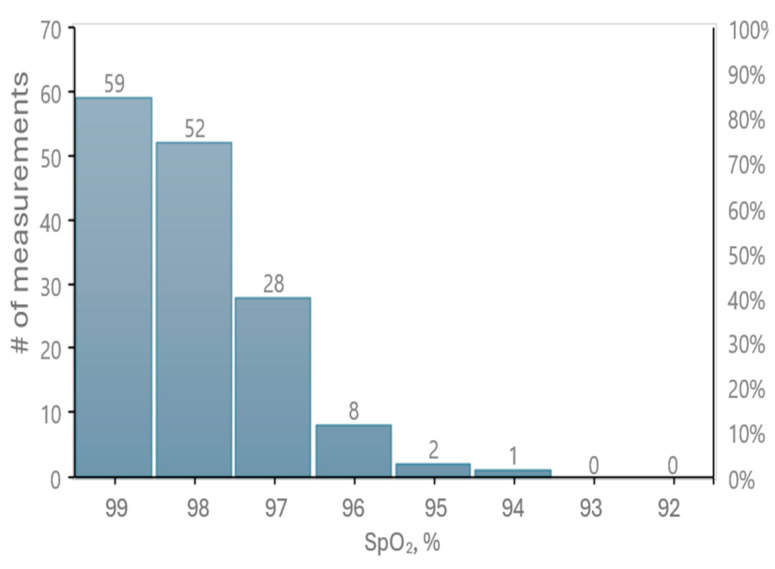
Distribution of *SpO*_2_ values recorded with the developed prototype oximeter during validation tests.

During testing, it was observed that the standard oximeter occasionally required additional time to generate measurement results. Consequently, the reference device failed to measure *SpO*_2_ values from the finger 23 times at 15 s and 2 times at 30 s. In contrast, the prototype successfully measured oxygen saturation at all intervals. Figure 9 presents the distribution of oxygen saturation values recorded by our prototype.

A statistical analysis of 275 cases within the *SpO*_2_ measurement range yielded an RMS (Root Mean Square) value of 0.26% for the difference between the reference readings and our tested oximetry device. Additionally, 94% of the measurement data fall within a 1% difference, while 98% show a difference of 2% or less in *SpO*_2_ values.

The results meet the accuracy requirements specified in the ISO 80601-2-61:2017 standard for *SpO*_2_ measurement, confirming that our device operates within the acceptable range. Therefore, the measurement accuracy of our prototype has been successfully validated.

## 5. Clinical Evaluation of the Prototype for Gingival Oxygen Saturation Measurement

The developed prototype was tested in real clinical conditions to evaluate its functional performance and ability to detect oxygen saturation levels in oral tissue.

Since the oximetry device is designed for use in the oral cavity, one of the most critical considerations is disinfection. In dentistry, the most common sources of infection stem from direct contact between instruments and a patient’s blood, saliva, and oral secretions. To mitigate infection risks, most dental devices are either disposable, immersed in strong disinfectants, sterilized in an autoclave, or used with disposable protective covers.

**Figure 10:**
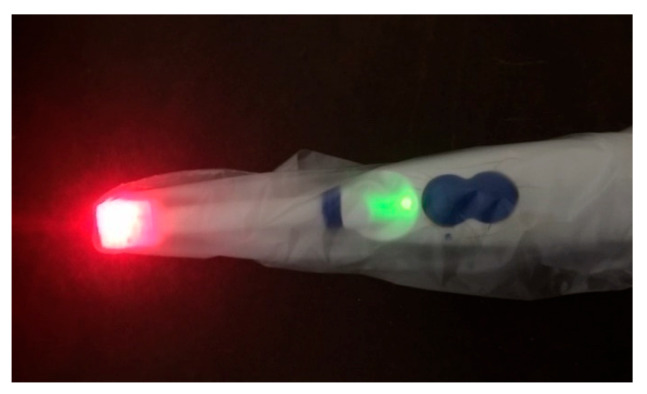
Disposable cover for the sensor probe protecting against infection.

For the developed oximetry prototype, the most effective solution to prevent cross-contamination and disease transmission during measurements is the use of a disposable cover over the sensor probe, as illustrated in Figure 10.

Experimental in vivo measurements were conducted at the Alexis Mendonça Clinic (Cascais, Portugal) on a randomly selected cohort of 17 patients aged 20 to 80 years. Ethical approval for this observational study was obtained from the Alexis Mendonça Clinic Ethics Committee (Decision No. 000112/2023, dated 14 March 2023). Written informed consent was obtained from all participants prior to their enrollment in the study. Measurements were carried out by an oral surgeon (medical dentist) during oral rehabilitation consultations.

**Figure 11:**
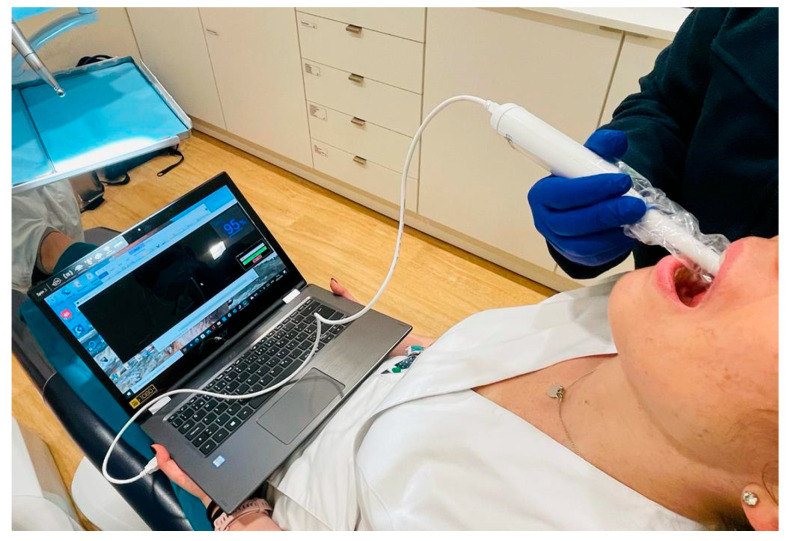
Field measurements of *SpO*_2_ in the gums in the clinic.

Oxygen saturation levels were measured symmetrically using the developed prototype, targeting the four quadrants of the buccal gingiva (Figure 11). The measurements were taken along the vestibular gum wall, progressing from the posterior molar region to the anterior incisor region in a sequential manner:

- 1st Quadrant;
- 2nd Quadrant;
- 3rd Quadrant;
- 4th Quadrant.

During the measurements, oxygen saturation levels were obtained with relative ease and remained stable at each measured point. The *SpO*_2_ values recorded for each quadrant are presented in Table 1.

The observed differences in oxygen saturation levels across quadrants may be attributed to variations in the blood vessel network. For instance, a 78-year-old female patient recorded *SpO*_2_ values of 95%, 93%, 94%, and 93% across the four quadrants. This patient had one implant per quadrant with prosthetic restorations but also exhibited periodontal issues in the 2nd and 4th quadrants, where oxygen saturation levels were lower. This reduction in *SpO*_2_ may be linked to impaired blood flow due to bacterial infection, which aligns with findings reported in the literature [47,48]. In some cases, edentulous areas (regions without teeth) that have been missing teeth for an extended period may experience reduced blood supply, as there are no natural teeth to support vascularization and nutrient exchange.

It should be emphasized that the primary objective of these measurements was to evaluate the performance of the developed device and its capability for in vivo assessment, rather than to establish definitive clinical diagnoses. For this reason, patient stratification according to oral health status and the corresponding statistical analysis of the obtained values were not performed in this small cohort. This can be considered a limitation of the present study, and larger-scale clinical trials are warranted to address this aspect.

Nonetheless, the preliminary trends observed among the participants provide valuable insights. Patients with a non-active form of periodontal disease and generally stable systemic health conditions (*n* = 9) demonstrated a mean gingival oxygen saturation level of 94.9%, with a standard deviation of 0.7 and a 95% confidence interval. In Table 1, this group includes one patient categorized as having “Implants” and the remaining individuals labeled as having a “Periodontal problem,” except for the 36-year-old patient who presented with active inflammatory periodontal disease and exhibited a mean *SpO*_2_ value of 93.0% across the four quadrants.

In contrast, patients identified in Table 1 as “Implants + Periodontal problem,” who presented with active periodontal disease and, in some cases, systemic health conditions (*n* = 8)—including cardiovascular disease, hypertension, type I and II diabetes, oncological disorders, and periodontal disease—exhibited a slightly lower mean gingival oxygen saturation level of 94.0% with a standard deviation of 0.6. Although the absolute difference between these groups is modest, it may reflect subtle physiological alterations in gingival microcirculation associated with systemic disease. This finding is further addressed in the Discussion section.

To date, reference values for gingival oxygen saturation have not been established, underscoring the need for further investigation.

## 6. Discussion

The developed prototype demonstrated good performance during testing, with high precision in measuring oxygen saturation. It exhibited good sensitivity and reliability, meeting the ISO 80601-2-61:2017 international standards for medical pulse oximetry devices.

The *SpO*_2_ values obtained from gingival measurements indicate that this technique has promising applications in dentistry, particularly for detecting abnormalities in tissues with impaired oxygenation.

Findings from these pilot measurements suggest the following potential clinical scenarios:

- Healthy gingiva: Higher oxygen saturation is expected due to adequate perfusion.
- Periodontal disease: Reduced gingival *SpO*_2_ may reflect impaired blood flow and inflammatory activity.
- Systemic correlations: Decreased gingival oxygenation may be linked to systemic conditions such as diabetes, cardiovascular disease, or anemia.

These findings are consistent with the growing body of evidence linking systemic health status to oral microvascular function and oxygenation [49,50]. Chronic conditions such as diabetes, cardiovascular disease, and periodontitis are known to impair vascular integrity, endothelial function, and local tissue oxygen delivery [51,52], which could explain some of the variations observed in this study. Although the present dataset is limited in size and scope, the results underscore the potential of gingival oxygen saturation monitoring as a complementary tool for early detection of systemic health influences on oral tissues.

The variations observed in oxygen saturation values across the four buccal quadrants should be interpreted with caution, as they remain within the expected accuracy limits of the prototype device and therefore cannot be considered clinically significant differences. Rather, these findings highlight the feasibility of performing consistent gingival oxygen saturation measurements at multiple intraoral sites.

Currently, there are no established normative reference values for gingival oxygen saturation in the literature. Typically, gingival *SpO*_2_ is lower than fingertip *SpO*_2_, owing to differences in blood perfusion. Therefore, it would be valuable to conduct simultaneous comparative measurements between healthy and diseased gum tissues.

The reported gingival *SpO*_2_ values in this preliminary study were within the range of 90–100%, which is consistent with expected peripheral oxygen saturation values. However, within this narrow range, it remains unclear whether systemic diseases (e.g., diabetes, hypertension, or pulmonary conditions) or local periodontal status can produce significant and clinically meaningful differences in gingival oxygenation. A limitation of this preliminary clinical evaluation is that systemic oxygen saturation (*SpO*_2_) was not simultaneously monitored using a validated device (e.g., fingertip pulse oximetry). As a result, potential variations in systemic oxygenation, particularly in patients with cardiovascular or pulmonary conditions, could not be accounted for. This may represent a confounding factor when interpreting gingival oxygen saturation values. Future studies will therefore incorporate systemic *SpO*_2_ monitoring to better contextualize gingival measurements and clarify the relationship between systemic health and oral oxygen saturation. Larger studies with improved calibration and systemic *SpO*_2_ monitoring will be needed to determine whether true physiological variations in regional gingival oxygenation exist.

Despite these limitations, gingival *SpO*_2_ monitoring offers valuable insights into microvascular health. Adequate oxygenation is a key indicator of healthy tissue perfusion, supporting essential oral health processes. For example, sufficient oxygen supply is critical for successful osseointegration after implant placement, as it ensures nutrient delivery, promotes new cell formation, and supports the establishment of a stable implant–bone interface. Likewise, in periodontal patients, maintaining proper oxygen levels is essential for controlling inflammation and stabilizing disease progression.

It should be noted that the device estimates oxygen saturation (*SpO*_2_) using the ratio-of-ratios method, which is largely independent of total blood flow and, therefore, does not capture changes in gingival perfusion. As a result, variations in local blood flow could represent a potential confounding factor in *SpO*_2_ estimation. Future iterations of the device may incorporate the Perfusion Index (PI) or similar parameters to complement oxygen saturation measurements and provide a more comprehensive assessment of gingival microcirculation.

In addition, inter-individual anatomical and pathological differences may influence the optical properties of the gingiva and, consequently, the accuracy of oxygen saturation measurements. For example, increased gingival thickness, inflammatory hyperemia, or edema associated with periodontitis may hinder light penetration and result in readings that are more representative of superficial capillary layers rather than deeper vascular structures. These factors should be carefully considered in future studies, and methodological strategies to account for them should be developed.

Although the sample size in this study was relatively small, limiting definitive clinical conclusions, the findings confirm the suitability of the developed device for assessing oxygen levels in the periodontal buccal gingiva. Future studies with larger and more diverse populations, standardized measurement protocols, and longitudinal follow-up will be necessary to explore their clinical implications for both preventive dentistry and systemic health monitoring [53,54].

An important consideration for the application of gingival reflectance-mode PPG is the limited penetration depth of light into oral soft tissues. While the prototype successfully detected gingival oxygen saturation, it remains uncertain whether the recorded signals reflect deeper periodontal microcirculation or are restricted mainly to superficial gingival layers. This limitation may restrict the comprehensive application of gingival PPG in assessing blood supply in periodontal and peri-implant tissues.

Clinical testing of the developed prototype highlighted several opportunities to further optimize both performance and usability. The most significant improvement involves enhancing ergonomics by refining the probe geometry and reducing its overall dimensions. These adjustments would increase the spatial resolution of site-specific measurements while improving suitability for intraoral use. At the same time, the current toothbrush-shaped probe demonstrated strong mechanical robustness, withstanding clinical handling while remaining comfortable and intuitive for dental practitioners.

Looking ahead, several key optimizations are planned for future iterations of the gingival oximetry probe:

- Wireless connectivity: Integrating wireless communication will enable seamless connection with computers, tablets, and mobile devices, supporting real-time monitoring and improved clinical workflow.
- Battery-powered operation: Incorporating a rechargeable, ultra-thin power source into the device body would eliminate the need for cable connections, thereby enhancing user comfort and patient mobility.
- Advanced user interface and data management: Developing a more sophisticated interface with expanded capabilities, such as saving, analyzing, and comparing results across multiple intraoral sites, will significantly enhance clinical utility. Integration of patient data storage will further support the creation of research databases, enabling large-scale studies and long-term monitoring.

By implementing these enhancements, the oximetry probe can evolve into a more advanced, user-friendly, and clinically valuable tool for assessing gingival and periodontal health. In parallel, future iterations will also integrate medical-grade power supplies, isolation measures, and other design refinements to ensure full compliance with IEC 60601-1 and ISO 80601-2 standards, thereby meeting both clinical usability and regulatory safety requirements.

## 7. Conclusions

Measuring oxygen saturation of the gingiva (gingival *SpO*_2_) represents a relatively novel approach in clinical practice. Unlike conventional pulse oximetry, which measures peripheral oxygen saturation (*SpO*_2_) at the fingertip or earlobe, gingival oximetry can provide localized insights into tissue oxygenation that may be relevant for dental, periodontal, and systemic health assessments.

A major challenge in developing pulse oximetry techniques for dentistry is the absence of standardized guidelines for measuring oxygen saturation in oral tissues. Extensive research is still required to establish baseline gingival *SpO*_2_ values across different intraoral sites, which would serve as a reference framework for both research and clinical applications.

From a clinical perspective, the prototype oximeter developed in this study has the potential to support prediction of healing outcomes and monitoring of the osseointegration process in dental implants. By assessing microvascularization through gingival oxygen measurements, the device could aid in treatment planning and patient follow-up.

Several limitations highlighted in this work must be addressed in future studies. These include technical improvements to ensure compliance with IEC 60601-1 safety standards, as well as the execution of larger-scale clinical trials to validate the clinical relevance of gingival oxygen saturation measurements. Pulse oximeters are simple, low-cost, non-invasive, non-radioactive, and accurate diagnostic tools. Their ease of use and favorable safety profile make them a promising technology for integration into routine dental practice.

## Figures and Tables

**Table 1:** Results for oxygen saturation in four quadrants at the buccal gingiva.

Patients Data		Clinical Information	*SpO*_2_, %			
Age	Gender		1st Q	2nd Q	3rd Q	4th Q
41	F	Periodontal problem	93	94	95	96
60	F	Implants + periodontal problem	94	95	94	95
27	F	Periodontal problem	94	95	94	95
43	F	Implants + periodontal problem	94	94	94	94
36	F	Periodontal problem	94	93	93	92
36	F	Periodontal problem	93	95	94	94
No, 21	F	Periodontal problem	95	96	96	95
28	F	Periodontal problem	96	95	96	96
51	M	Implants + periodontal problem	95	96	94	95
78	M	Implants + periodontal problem	93	94	95	94
74	F	Implants	95	94	95	95
30	M	Periodontal problem	96	97	96	95
43	F	Periodontal problem	95	94	95	95
52	F	Implants + periodontal problem	94	94	93	94
78	F	Implants + periodontal problem	95	93	94	93
23	M	Periodontal problem	94	94	95	94
55	F	Implants + periodontal problem	94	93	94	94

## Data Availability

The raw data supporting the conclusions of this article will be made available by the authors on request.
